# Implant Pocket Plane Selection in Primary Breast Augmentation: A Meta-Analysis and Systematic Review of Complication Profiles

**DOI:** 10.1007/s00266-026-05706-5

**Published:** 2026-02-26

**Authors:** Raed Alderhali, Wameth Alaa Jamel, Sina Dehnadi, Ishith Seth, Ankur Khajuria

**Affiliations:** 1 Department of Medicine, Newcastle University, Newcastle upon Tyne, UK; 2 Al-Wasity Teaching Hospital, Baghdad, Iraq; 3 Royal Free London NHS Foundation Trust, London, UK; 4 Department of Plastic and Reconstructive Surgery, Peninsula Health, Melbourne, VIC Australia; 5 Department of Plastic Surgery, Imperial College Healthcare NHS Trust, London, UK

**Keywords:** Breast augmentation, Implant pocket plane, Submuscular, Subglandular, Subfascial, Dual-plane, Capsular contracture

## Abstract

**Background:**

Implant pocket selection is a key determinant of safety and aesthetic outcomes in primary breast augmentation. Despite the widespread use of subglandular, subfascial, submuscular, and dual-plane techniques, no clear consensus exists on the optimal approach.

**Methods:**

A PRISMA-guided systematic review and meta-analysis of PubMed, Embase, Scopus, and Web of Science identified studies on implant pocket-related complications in primary breast augmentation from inception to February 2025. One-arm and pairwise random-effects meta-analyses were performed, with heterogeneity assessed by *I*^2^, publication bias by Egger’s test, and risk of bias by ROBINS-I.

**Results:**

Ninety one studies (51,524 patients; mean age 33.0 years, BMI 21.6 kg/m^2^) were included. Submuscular placement was most common (*n* = 22,764), followed by dual-plane (15,480), subglandular (7,870), and subfascial (5,410). Capsular contracture (CC) was highest with subglandular implants (6.85%) compared to subfascial (2.80%), dual-plane (1.99%), and submuscular (1.83%) implants. Pairwise analysis revealed an increased CC risk with subglandular versus submuscular placement (RR = 2.84; *p* = 0.041) and a protective effect of subfascial versus subglandular placement (RR = 0.24; *p* = 0.013). Hematoma did not differ between subglandular and submuscular (RR = 0.88; *p* = 0.70) but was lower with subfascial versus subglandular (hematoma (RR = 0.21; 95% CI 0.02–2.31; *p* = 0.2004; *I*^2^ = 65.3%) although this failed to reach statistical significance. Other complications were infrequent: seroma (0.5–1.4%), infection (0.5–1.0%), reoperation (2.3–4.1%), and displacement (0.8–1.7%).

**Conclusions:**

All pocket planes demonstrated low complication rates with notable variation. Submuscular and dual-plane showed the most favorable profiles, while subfascial may represent a balanced alternative. These findings may help refine surgical decision-making, offering tailored pocket selection strategies to optimize both safety and aesthetic outcomes in primary breast augmentation.

**Level of Evidence III:**

This journal requires that authors assign a level of evidence to each article. For a full description of these Evidence-Based Medicine ratings, please refer to the Table of Contents or the online Instructions to Authors www.springer.com/00266.

**Supplementary Information:**

The online version contains supplementary material available at 10.1007/s00266-026-05706-5.

## Introduction

Breast augmentation remains one of the most frequently performed procedures in aesthetic plastic surgery [1]. Over recent decades, various surgical techniques have been developed for implant pocket placement, each with specific advantages and disadvantages [2–4]. The four primary approaches, namely the subglandular, subfascial, submuscular, and dual-plane, remain the cornerstone of aesthetic breast augmentation, each with distinct anatomical considerations, benefits, and drawbacks [5].

The subglandular plane, with the implant placed above the muscle and pectoral fascia, offers faster recovery but is linked to higher rates of capsular contracture and implant visibility, especially in patients with minimal soft tissue coverage [6]. The subfascial technique positions the implant beneath the pectoral fascia, potentially offering better coverage and reduced capsular contracture risk compared to subglandular placement, without the animation deformities seen in submuscular techniques. In the submuscular approach, the implant lies beneath the pectoralis major muscle, providing greater soft tissue coverage and a lower capsular contracture rate, albeit at the cost of increased postoperative pain and a higher risk of animation [6]. The dual-plane method, a modification of the submuscular approach, partially releases the muscle to improve lower pole expansion while preserving upper pole coverage, aiming to combine aesthetic flexibility with a favorable safety profile. [7]

Despite the widespread use of all four pocket planes, there is no clear consensus on which technique offers the most favorable balance between safety and aesthetic outcomes. Existing literature varies widely in reported complication rates, and a comprehensive, comparative synthesis has been lacking. To address this, we performed a systematic review and meta-analysis to directly evaluate and compare the complication profiles associated with subglandular, subfascial, submuscular, and dual-plane implant placement in primary breast augmentation.

## Methods

The study protocol was prospectively registered on PROSPERO [8]. As this review synthesizes previously published data, institutional review board approval and informed consent were not required. The authors report no conflicts of interest.

A comprehensive search of Medline (PubMed), Embase, Scopus, and Web of Science was performed from database inception to February 2025 the following search string was utilized in the search ((breast augmentation) OR (breast implant) OR (augmentation mammaplasty)) AND ((subglandular) OR (submuscular) OR (dual plane) OR (subfascial)). Two independent reviewers conducted the search, supplemented by title–abstract screening and manual review of reference lists. No language, date, or article-type restrictions were applied. The review followed PRISMA 2020 guidelines. [9]

### Study Selection

**Fig. 1:**
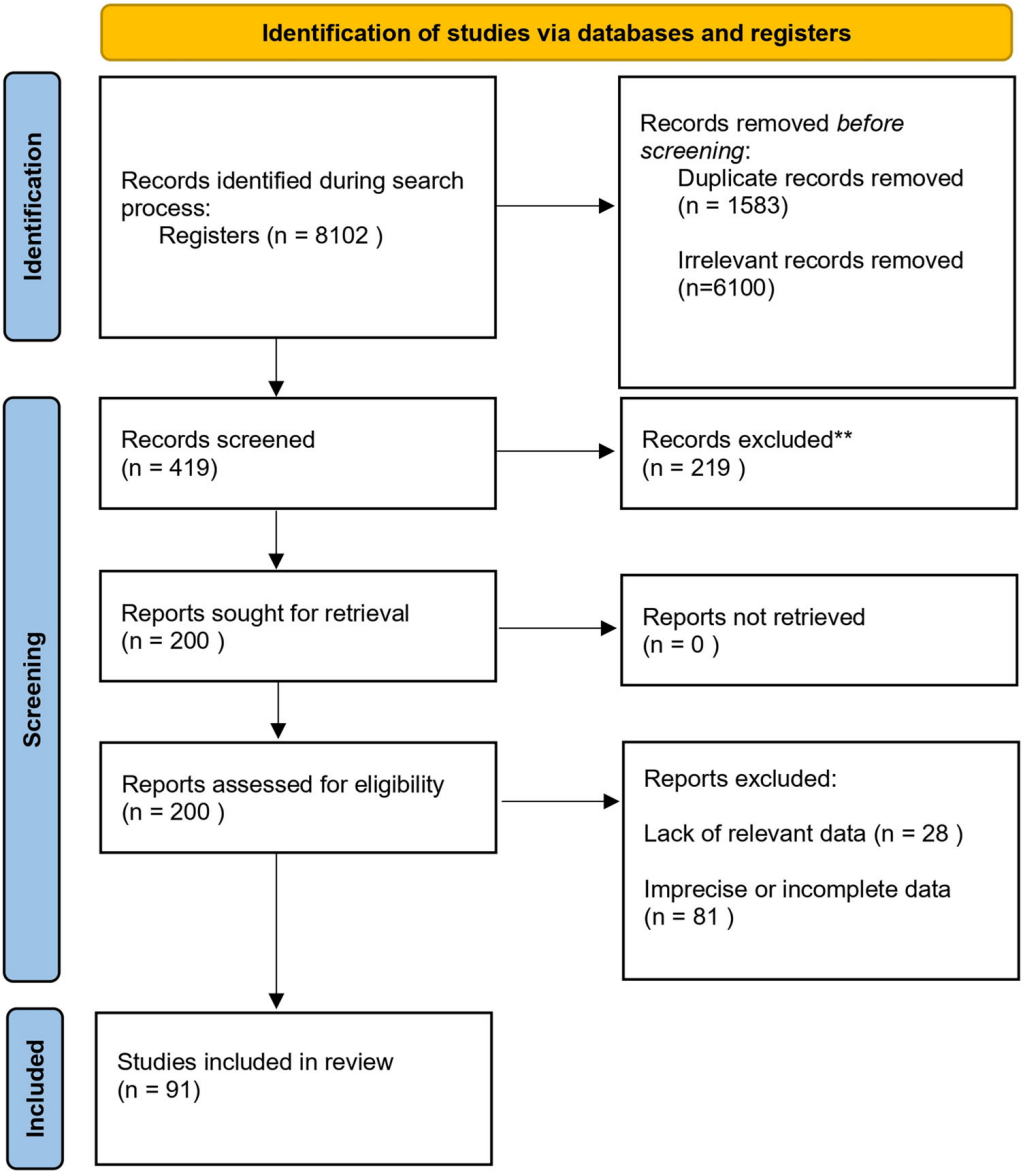
PRISMA flowchart showcasing study selection process

Eligible studies included randomized controlled trials, prospective or retrospective cohort studies, and case series with >20 patients undergoing primary implant-based breast augmentation with clearly specified implant pocket plane. Studies had to report extractable numerical data on postoperative complications. Exclusion criteria were case reports, reviews, conference abstracts, letters, studies with incomplete or irrelevant data, revision procedures, concomitant mastopexy, and reconstructive cohorts. Discrepancies in study selection were resolved by consensus. The PRISMA flow diagram is presented in Fig. 1.

### Data Extraction

Two reviewers independently extracted data on study characteristics (sample size, patient demographics, study design, follow-up duration, implant type and surface, incision and drain use) and reported complications (e.g., capsular contracture, hematoma, seroma, infection, displacement, rippling, palpability, reoperation). When needed, authors of primary studies were contacted for clarification. The primary outcomes were the pooled complication rates for each implant pocket plane and pairwise comparisons where available. For capsular contracture, only studies with ≥12 months of follow-up were included. Because reporting intervals varied widely, Baker grade III/IV contracture at the latest available follow-up was used for analysis.

### Risk-of-Bias Assessment

Risk of bias in non-randomized studies was evaluated using ROBINS-I V2 [10] and case series were appraised using the Joanna Briggs Institute checklist. All assessments were performed independently by two reviewers, with disagreements resolved by discussion. Publication bias was explored through visual inspection of funnel plots and Egger’s regression test.

### Statistical Analysis

Random-effects meta-analyses were performed for each complication, with pairwise random-effects models used for direct comparisons between pocket planes. Proportions were transformed using the Freeman–Tukey method and back-transformed for reporting. DerSimonian–Laird τ^2^, Hartung–Knapp confidence intervals, and a continuity correction of 0.5 for zero-event studies were applied. Heterogeneity was assessed using Cochran’s Q and *I*^2^ statistics. Analyses were conducted in R (v4.3.0).

## Results

A total of 91 studies encompassing 51,524 patients were included. Submuscular placement was the most common pocket (*n* = 22,764), followed by dual-plane (*n* = 15,480), subglandular (*n* = 7,870), and subfascial (*n* = 5,410). The pooled mean age was 33.0 years (SD 4.21), and the mean BMI was 21.6 kg/m^2^ (SD 0.87). The dataset consisted of two randomized controlled trials, 22 retrospective cohort studies, 17 prospective cohort studies, 35 retrospective case series, and 15 prospective case series. Implant volume did not differ significantly across planes (*p* = 0.21), although submuscular placement trended toward slightly larger volumes. Implant surface type varied significantly (χ^2^(12) = 22.33, *p* = 0.034): subfascial implants were exclusively textured; dual-plane cohorts demonstrated mixed surface types; and submuscular and subglandular cohorts differed in their relative use of smooth, textured, and polyurethane devices. Surface distribution differed significantly between subfascial and both dual-plane (*p* = 0.0496) and submuscular (*p* = 0.0189) placement. Incision type did not differ significantly (*p* = 0.70), though dual-plane procedures primarily used an inframammary approach, while subfascial cases were more evenly split between inframammary and transaxillary incisions. Drain usage varied significantly (*p* = 0.03), with the highest rate in subfascial placement and none reported in subglandular cases. Full study characteristics are provided in Supplementary Digital Content 1, with the complete list of included studies available in Supplementary Digital Content 2.

Across pocket planes, complication rates were generally low. Subglandular placement demonstrated the highest pooled incidence of capsular contracture (6.9%, 95% CI 1.7–24.2%) and a reoperation rate of 2.8% (95% CI 1.4–5.6%), while other complications occurred infrequently and are detailed in Table 1. Subfascial placement showed a lower capsular contracture rate of 2.80% (95% CI 1.04–7.34%) and a reoperation rate of 2.34% (95% CI 0.90–5.45%), with all other events similarly uncommon (Table 2). Submuscular augmentation had the lowest capsular contracture incidence at 1.8% (95% CI 1.13–2.96%) but demonstrated the highest reoperation rate at 4.1% (95% CI 2.35–7.15%); remaining complications were generally reported at rates below 1–2% (Table 3). Dual-plane placement showed a capsular contracture rate of 1.99% (95% CI 1.53–2.58%) and a reoperation rate of 2.39% (95% CI 1.48–3.86%), with other complications occurring rarely (Table 4). Full pooled estimates for all secondary complications are presented in the corresponding tables.

**Table 1:** Statistical results regarding the pooled incidence of complications in the subglandular plane

Subglandular Plane							
Complication	N	Pooled incidence (%)	LCI (%)	HCI (%)	*I*^2^	Q	Eggers test (z)
Capsular contracture	2093	6.9	1.7	24.2	1	180.53	− 1.2444
Infection	1574	1.0	0.6	1.6	0	6.49	− 0.9621
Asymmetry	86	0.5	0.2	1.2	0	7.58	− 1.0284
Displacement	1181	1.5	0.7	3.2	0.6	24.57	− 3.0176
Hematoma	1624	1.2	0.7	2.4	0.4	15.73	− 1.6915
Hyposthesia	39	0.6	0.2	2.1	0.8	38.72	− 5.6338
Palpability	741	1.1	0.3	4.0	0.9	131.58	− 1.9493
Reoperation	1379	2.8	1.4	5.6	0.8	52.67	− 2.9497
Rippling	741	0.6	0.2	1.4	0	7.56	− 1.3168
Wound complications	1191	1.0	0.5	1.9	0.1	10.43	− 1.3781
Seroma	1002	1.4	0.7	2.8	0.5	16.59	− 2.6689

*LCI* lower confidence interval, *HCI* higher confidence interval, *Q* Cochran’s Q

**Table 2:** Statistical results regarding the pooled incidence of complications in the subfascial plane

Subfascial Plane							
Complication	N	Pooled incidence (%)	LCI (%)	HCI (%)	*I*^2^	Q	Eggers test (z)
Capsular contracture	3554	2.8	1.04	7.34	1	259.34	− 3.2384
Infection	2417	0.6	0.35	0.89	0	6.17	− 0.1988
Asymmetry	367	0.8	0.27	2.49	0.8	46.05	− 5.4289
Displacement	2494	0.8	0.33	1.87	0.6	30.39	− 2.2402
Hematoma	3506	1	0.59	1.67	0.3	14.75	− 0.898
Hyposthesia	152	0.5	0.18	1.54	0.7	31.8	− 4.3589
Palpability	1503	0.6	0.2	1.4	0.6	24.84	− 0.8159
Ptosis	110	0.4	0.18	1.11	0.1	16.08	− 2.066
Reoperation	2647	2.3	0.99	5.45	0.8	58.31	− 7.1967
Rippling	2432	0.8	0.32	2.01	0.7	40.41	− 1.6835
Rupture	1032	0.7	0.30	1.47	0.4	17.91	− 3.1247
Wound complications	2596	1.8	0.73	4.32	0.8	66.36	− 2.3839
Seroma	2585	1.0	0.37	2.94	0.9	87.51	− 2.5005

*LCI* lower confidence interval, *HCI* higher confidence interval, *Q* Cochran’s Q

**Table 3:** Statistical results regarding the pooled incidence of complications in the submuscular plane

Submuscular Plane							
Complication	N	Pooled incidence, (%)	LCI (%)	HCI (%)	*I*^2^	Q	Eggers test (z)
Capsular contracture	10801	1.8	1.13	2.96	0.9	207.94	− 3.3444
Infection	5278	0.5	0.27	0.89	0.6	72.4	− 2.7728
Asymmetry	2579	0.5	0.24	1.00	0.8	129.5	− 5.5157
Displacement	10392	1.7	0.95	3.01	0.9	235.27	− 4.3125
Hematoma	9601	0.8	0.6	1.18	0.5	49.96	− 2.308
Hyposthesia	2894	0.5	0.22	1.06	0.9	241.05	− 6.2702
Palpability	1790	0.3	0.16	0.48	0.3	37.77	− 1.9745
Ptosis	2915	0.6	0.27	1.23	0.9	257.65	− 7.0256
Reoperation	16257	4.1	2.35	7.15	1	619.66	− 5.4524
Rippling	3970	0.5	0.25	1.01	0.9	188.81	− 4.6869
Rupture	9757	1.1	0.68	1.85	0.7	104.56	− 7.9117
Wound complications	5255	0.9	0.49	1.54	0.7	84.57	− 6.3344
Seroma	4969	0.5	0.25	0.87	0.7	84.66	− 3.4084

*LCI* lower confidence interval, *HCI* higher confidence interval, *Q* Cochran’s Q

**Table 4:** Statistical results regarding the pooled incidence of complications in the dual-plane

Dual-Plane							
Complication	N	Pooled incidence (%)	LCI(%)	HCI(%)	*I*^2^	Q	Eggers test (z)
Capsular contracture	11115	1.99	1.53	2.58	0.7	84.76	− 1.8337
Infection	5889	0.50	0.35	0.72	0	29.03	− 1.2725
Asymmetry	1858	0.6	0.28	1.23	0.9	279.05	− 5.9585
Displacement	6111	1.3	0.77	2.13	0.8	120.18	− 7.7984
Hematoma	8053	0.9	0.75	1.15	0	29.93	− 1.3255
Hyposthesia	392	0.4	0.19	0.74	0.7	104.96	− 4.5266
Palpability	1222	0.4	0.22	0.94	0.8	159.57	− 7.0351
Ptosis	333	0.3	0.16	0.43	0.4	23.74	− 0.0096
Reoperation	7584	2.4	1.48	3.83	0.8	183.85	− 5.4929
Rippling	420	0.4	0.20	0.67	0.1	32.24	− 4.0098
Rupture	6897	0.5	0.37	0.67	0	23.18	− 0.6081
Wound complications	3575	0.8	0.4	1.54	0.8	136.53	− 4.9713
Seroma	7514	0.7	0.46	0.92	0.1	33.59	− 1.9151

*LCI* lower confidence interval, *HCI* higher confidence interval, *Q* Cochran’s Q

**Fig. 2:**
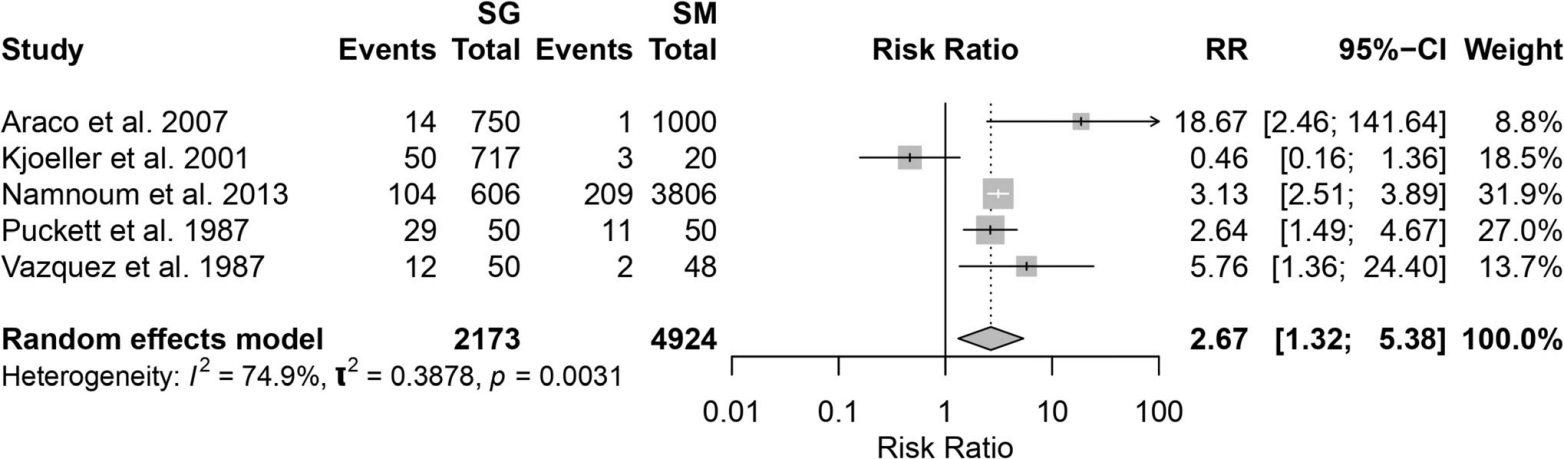
Forest plot comparing the incidence of capsular contracture between subglandular (SG) and submuscular (SM) implant placement

**Fig. 3:**
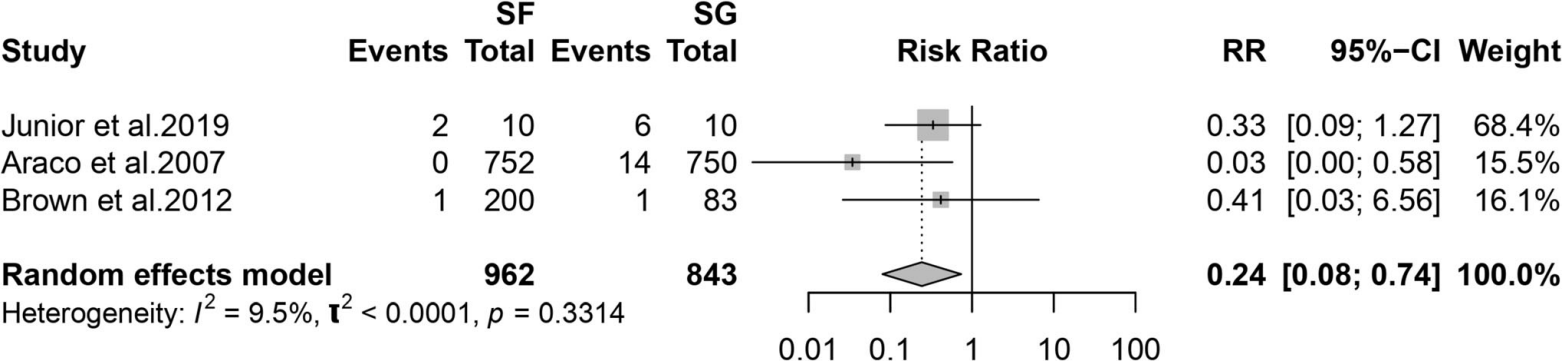
Forest plot comparing the incidence of capsular contracture between subfascial (SF) and subglandular (SG) implant placement

Pairwise comparisons showed that subglandular placement carried a significantly higher risk of capsular contracture than submuscular placement (RR = 2.84; 95% CI 1.05–7.72; *p* = 0.0407; *I*^2^ = 74.6%) (Fig. 2), while hematoma rates did not differ between these planes (RR = 0.88; 95% CI 0.45–1.71; *p* = 0.7005; *I*^2^ = 0%). Compared with subglandular placement, subfascial implants demonstrated a significantly lower risk of capsular contracture (RR = 0.24; 95% CI 0.08–0.74; *p* = 0.013; *I*^2^ = 9.5%) (Fig. 3). Subfascial placement also showed nonsignificant trends toward lower reoperation (RR = 0.12; 95% CI 0.004–3.72; *p* = 0.2276; *I*^2^ = 81.4), hematoma (RR = 0.21; 95% CI 0.02–2.31; *p* = 0.2004; *I*^2^ = 65.3%), and rippling (RR = 0.32; 95% CI 0.003–29.44; *p* = 0.6200; *I*^2^ = 85.1), although all analyses demonstrated substantial heterogeneity.

### ROBINS-I

ROBINS-I assessment showed considerable variability in study quality. Overall, 9 studies (22%) were judged at low risk of bias, 14 (34%) at moderate risk, and 18 (44%) at serious risk. Risk of bias was generally low for intervention classification and deviations from intended interventions. For participant selection, 27 studies (66%) were rated low risk, 12 (29%) moderate risk, and 2 (5%) serious risk. Confounding represented the major limitation, with only 10 studies (24%) rated low risk and 18 (44%) judged at serious risk. Most studies had low risk of bias related to missing data (31 studies, 76%) and outcome measurement (18 studies, 44% low risk; 23 studies, 56% moderate risk). Selective reporting was uncommon, with 40 studies (98%) rated at low risk.

### JBI Checklist for Case-Series

The methodological quality of the 50 case series, assessed using the Joanna Briggs Institute checklist, was generally high. Most studies demonstrated clear inclusion criteria, reliable measurement methods, appropriate participant inclusion, and adequate reporting of clinical information and site characteristics. Complete inclusion of eligible participants was reported in 47 studies (94%), and participant demographics were clearly described in 40 studies (80%). Outcome reporting was clear in 49 studies (98%). Appropriate statistical analysis was performed in 39 studies (78%), while it was not applicable in 11 studies. Overall, the case series demonstrated minimal risk of bias across most domains.

## Discussion

Primary breast augmentation remains one of the most commonly performed aesthetic procedures worldwide, and surgical technique continues to evolve in response to shifting aesthetic preferences, implant technology, and efforts to minimize complications [11]. A key technical decision during augmentation involves the selection of the implant pocket plane, which can substantially influence both short- and long-term outcomes. While subglandular, subfascial, submuscular, and dual-plane techniques are all routinely employed, consensus remains lacking regarding which plane offers the most favorable balance between safety, aesthetics, and complication risk [12].

This meta-analysis demonstrated meaningful differences in complication profiles across pocket planes, particularly with respect to capsular contracture. The highest pooled incidence was observed in subglandular placement, followed by subfascial, dual-plane, and submuscular. Pairwise comparisons further confirmed that subglandular placement significantly increased the risk of capsular contracture compared to submuscular implants, while subfascial placement offered a significant protective effect compared to subglandular. These findings reinforce a longstanding trend reported in earlier literature, showcasing that the submuscular plane is associated with lower rates of capsular contracture. Multiple mechanisms have been proposed to explain the reduced risk of capsular contracture associated with submuscular implant placement [13]. The pectoralis major muscle not only provides improved implant coverage and mechanical buffering, but also helps preserve local vascularity, which may reduce infection risk and subsequent fibrotic response [14]. Notably, our analysis suggests that implant plane exerts a more substantial influence on CC than surface texture, with submuscular placement showing the lowest overall incidence of CC despite containing the highest proportion of smooth implants. This finding is corroborated by Lista et al., who observed that implant surface alone did not significantly affect CC rates in a large subglandular cohort, instead emphasizing meticulous surgical technique aimed at minimizing contamination as the more decisive factor relative to device characteristics [15]. At the same time, it should be acknowledged that all subfascial cases in our dataset involved textured implants, whereas subglandular cohorts included a heterogeneous mix of textured, smooth, and polyurethane devices. This imbalance raises the possibility that the lower CC rates observed with subfascial placement may, at least in part, reflect an implant-surface effect rather than a purely plane-related advantage. Future comparative studies that standardize implant surface across pocket planes are necessary to clarify whether the apparent protective effect of the subfascial technique is intrinsic to the plane itself, or confounded by device selection.

Hematoma was infrequent across all planes, ranging from 0.84% in submuscular to 1.24% in subglandular placement, pairwise analysis demonstrated a non-statistically significant reduction in hematoma risk with subfascial compared to subglandular placement (RR = 0.21; 95% CI 0.02–2.31; *p* = 0.20). The etiology of hematoma may relate to surgical dissection technique, implant pocket vascularity, and intraoperative hemostasis. Submuscular and subfascial placements potentially reduce hematoma through more confined pocket dissection and muscular compression of the implant bed, although this remains speculative [16].

Reoperation is a crucial metric in evaluating long-term outcomes in breast augmentation, reflecting both complication management and patient-driven revisions for aesthetic reasons. Interestingly, submuscular placement, despite offering the lowest CC incidence, demonstrated the highest pooled reoperation rate (4.13%), potentially reflecting aesthetic concerns such as animation deformity or implant malposition, which submuscular implants also had the highest incidence of (1.69%). Dual-plane and subglandular placements showed similar intermediate rates, while subfascial had the lowest (2.34%). These findings highlight that patient-driven aesthetic revisions may influence reoperation as much as traditional complications.

This meta-analysis re-emphasizes that when determining the optimal pocket plane and implant type, surgeons should individualize their approach to the patient’s anatomy, soft-tissue characteristics, and aesthetic goals. Patients with thin soft tissue envelopes or minimal glandular coverage are best served with submuscular or dual-plane placement, which reduces implant visibility and capsular contracture risk. Subfascial placement may be an attractive alternative in patients with moderate tissue coverage, as it provides additional support without the risk of animation deformity inherent to submuscular techniques. Subglandular augmentation should be reserved for carefully selected patients with adequate parenchymal thickness and minimal concern for contracture. Implant selection should also align with patient-specific factors: smooth implants may be preferable in submuscular pockets where contracture risk is lowest. At the same time, textured or polyurethane-coated devices may provide benefit in subfascial or subglandular planes where coverage is reduced. A thorough preoperative assessment, including evaluation of pinch thickness, skin quality, breast footprint, and chest wall anatomy, remains essential for tailoring both pocket choice and implant type to achieve safe, durable, and aesthetically pleasing outcomes.

Furthermore, although subpectoral placement has long been associated with lower capsular contracture rates, the emerging literature on rigorous contamination control has primarily examined surface (textured vs smooth) rather than the pocket plane. Notably, recent evidence suggests that the apparent advantage of textured devices may attenuate when standardized protocols (e.g., the 14-point plan) are applied, but whether a similar attenuation occurs for plane choice remains untested. [15, 17] Our aim is not to dispute the protective association historically attributed to subpectoral placement, but to contextualize it. In contemporary practice where contamination is uniformly minimized, the magnitude of any plane-related benefit may differ from legacy estimates. Accordingly, a valuable next step would be prospective studies comparing subpectoral and subglandular cohorts under harmonized contamination-control protocols (ideally with patient allocation guided by baseline risk factors, and either restricting to a single surface type or prespecifying adjustment for surface) [14, 18]. Such a design would isolate the true effect of pocket selection on capsular contracture risk, disentangle it from surface characteristics and perioperative technique, and clarify whether a residual plane-specific advantage persists, and to what extent. [19]

This study has several limitations. Significant heterogeneity was observed across pooled analyses, reflecting variation in surgical technique, implant characteristics, follow-up, and reporting standards; despite random-effects modeling, this reduces the precision of estimates. Although we restricted inclusion to studies with at least 12 months of follow-up, the actual duration was highly variable, ranging from 1 year in some series to over a decade in registry data, and reporting of capsular contracture at interim time points was inconsistent. We therefore relied on the latest available follow-up from each study, which may have introduced variability in outcome ascertainment. Methodological quality also varied by study design: while case series scored highly on the JBI checklist due to clear inclusion criteria and consistent reporting, comparative non-randomized studies frequently showed moderate to serious risk of bias on ROBINS-I, particularly from confounding. This limits the strength of comparative conclusions regarding implant plane outcomes, even if descriptive insights from case series remain valuable. Furthermore, rare complications such as rupture or severe infection were inconsistently reported, leading to wide confidence intervals. Patient-related variables (age, BMI, gender identity) and procedural details (incision type) could not be analyzed uniformly, limiting subgroup analyses. In addition, no head-to-head comparisons or pairwise analyses were possible for the dual-plane technique, as no studies provided direct comparative data for this pocket. Another important limitation relates to the broad temporal range of included studies, spanning more than 25 years. During this time, implant technology and surgical techniques have evolved substantially. While narrowing the inclusion window to contemporary studies might have reduced heterogeneity, it would have substantially limited statistical power and reduced the ability to assess rare complications. We therefore choose to retain a wide timeframe, recognizing that this enhances comprehensiveness at the cost of increased clinical and methodological variability. Finally, the predominance of retrospective designs raises concern for selection and reporting bias, and publication bias cannot be excluded. Accordingly, these findings should be interpreted as reflecting overall trends in complication rates rather than precise incidence values. Prospective, standardized studies are needed to more definitively define complication risk profiles and guide evidence-based pocket selection in breast augmentation.

## Conclusions

Pocket selection meaningfully influences outcomes in primary augmentation. Submuscular and dual-plane placements show lower capsular contracture than subglandular, while subfascial appears intermediate and had the lowest reoperation in pooled estimates. These advantages must be weighed against trade-offs, including animation and malposition with submuscular pockets. Given heterogeneity and bias across studies, decisions should be individualized to anatomy, soft-tissue coverage, and patient goals. Emerging contamination-control protocols appear to attenuate surface-related differences; whether analogous attenuation applies to plane choice remains unknown. Prospective, standardized trials controlling for contamination and surface are needed to isolate plane effects and refine evidence-based selection.

## Supplementary Information

Below is the link to the electronic supplementary material.

Supplementary file1 (DOCX 434 kb)

Supplementary file2 (DOCX 25 kb)
