# Supplementary material for: Implant Pocket Plane Selection in Primary Breast Augmentation: A Meta-Analysis and Systematic Review of Complication Profiles
Source: Aesthetic Plast Surg. 2026 Feb 26;50(11):3979–87. doi: 10.1007/s00266-026-05706-5 (PMC13315495; doi:10.1007/s00266-026-05706-5)
Supplement: Supplementary file 1 — Supplementary file1 (DOCX 434 kb) [file 266_2026_5706_MOESM1_ESM.docx]

| **Study** | **Year** | **Type of study** | **Number of Patients** | **Surgical Plane** | **Incision Type** | **Implant Type** | **Implant Surface** | **Mean Implant Size (cc)** | **Implant Size Range (cc)** |
| --- | --- | --- | --- | --- | --- | --- | --- | --- | --- |
| Aboelatta et al. | 2015 | Prospective | 96 | Subglandular | Inframammary | Round, High Profile, Cohesive Gel (Mentor, POLYTECH) | Textured | Group 1: <300, Group 2: 300-400, Group 3: 400-600 | 275–600 |
| Adams et al. | 2006 | Prospective | 165 | Dual plane (Type 1) | Not specified | Saline (76%), Silicone Gel (24%) | Smooth (78%), Textured (22%) | NS | NS |
| Adams et al. (2008) | 2008 | Prospective | 300 | Dual plane (Type 1) | Inframammary (98%) | Saline (128), Form-Stable Cohesive Gel (172) | Smooth Round (most saline), Textured (all gel) | 289 overall; 302 (saline), 276 (gel) | 150–560 |
| Alderman et al. | 2023 | Prospective, multicenter clinical trial (GLOW) | 151 | Submuscular | Inframammary (77.5%), Periareolar (7.9%), Other (13.9%) | MemoryGel Xtra Silicone Gel | Mostly Smooth (95.4%) | Median 410 cc | 240–790 |
| Araco et al. | 2007 | Retrospective Case series | 3002 | Subglandular (25%) vs Subfascial (25%) vs Submuscular (33.3%) vs Dual Plane (16.7%) | Inframammary (94.1%), Periareolar (5.9%) | Silicone gel (Eurosilicone, Mentor, PIP), round/anatomic | Textured | 296.8 ± 46.6 | NS |
| Asplund et al. | 1996 | Prospective | 61 | Submuscular | Periareolar | saline | Textured | 250 | NS |
| Aygit et al. | 2012 | Prospective | 27 | Subfascial | Transaxillary | Anatomical, textured, cohesive gel (Cohesive III, Mentor) | Textured | 235 | 180–300 |
| Barbato et al. | 2004 | Prospective | 110 | Subfascial | Periareolar | Silicone gel | 85% Textured, 15% Smooth | 300–350 most common | 195–450 |
| Batiukov et al. | 2024 | Retrospective Cohort | 333 | Dual plane | Periareolar or Inframammary (exact distribution not fully quantified) | Polyurethane-covered silicone implants | Polyurethane | NS | NS |
| Bengtson et al. | 2007 | Prospective, Multicenter Clinical Trial | 492 | Submuscular | Inframammary (86.8%), Periareolar (12.2%), Axillary (0.4%) | Style 410 Highly Cohesive Silicone Gel | Textured (Biocell) | NS | NS |
| Bletsis et al. | 2025 | Retrospective Case Series | 716 | Dual plane (Type 2) | Inframammary | Nagor and Perle round silicone implants | Smooth and microtextured | 305 (range: 140–630) | 140–630 |
| Blount et al. | 2013 | Retrospective Case series | 856 | Submuscular | Mixed (Inframammary, Transaxillary) | Silicone | Mixed (Smooth & Textured) | Approx. 350 | 150–600 |
| Bolletta et al. | 2019 | Prospective | 300 | Submuscular | 4-6 cm incision, not formally specified | Silicone | Textured | NS | 225–420 |
| Bosch et al. | 2002 | prospective | 200 | Dual plane (Type 2) | Inferior hemiareolar | Silicone gel | Smooth | NS | 160–340 |
| Brown et al. | 2005 | Retrospective Case series | 118 | Submuscular | Inframammary (95), Circumareolar (17), Periareolar (5), Inverted teardrop (1) | Inamed Style 410 anatomical cohesive gel | Textured | NS | NS |
| Brown et al. | 2012 | Retrospective Case series | 283 | Subfascial (70%) vs Subglandular (30%) | Inframammary | Silicone | Textured | NS | 240–620 |
| Bruck et al. | 1990 | Retrospective Case series | 63 | Subglandular | Inframammary | Ashley Natural-Y polyurethane-covered silicone | Polyurethane | NS | NS |
| Chatterjee et al. | 2020 | Retrospective Case series | 111 | Subfascial | Periareolar or Inframammary | Silicone | 72 textured, 39 smooth | 350 | 180–550 |
| Chiemi et al. | 2022 | Retrospective Cohort | 133 | Dual plane | Inframammary (100%) | Silicone gel (Allergan, Sientra, Mentor; round and shaped) | Smooth (63.2%), Micro-textured (36.8%) | Smooth: 435, Micro-textured: 411 | NS |
| Coleman et al. | 1991 | Prospective | 50 | Subglandular | Inframammary | Silicone | Textured | NS | NS |
| Dancey et al. | 2012 | Retrospective Case series | 1400 | Subfascial | Inframammary (5 cm) | Silicone (GFX, Eurosilicone, McGhan, Silimed, MistiGold, Siltex) | Textured | NS | 195–800 |
| Diaz et al. | 2017 | Retrospective Case series | 494 | Dual plane (Type 1) | Inframammary (92%), Periareolar (8%) | Smooth round silicone (56%), Saline (41.5%), Shaped silicone (2.5%) | All round implants smooth; shaped implants may be textured | 380 | 176–700 |
| Fanous et al. | 2004 | Retrospective Case series | 319 | Submuscular | Inframammary | Mentor Style 1600 smooth saline | Smooth | Most common: 300 mL (40.1%), then 350 mL (21.6%) | 150–425 |
| Govrin-Yehudain et al. | 2021 | Retrospective Case series | 655 | Subglandular | Inframammary (95.7%), Circumvertical (4.3%) | B-Lite silicone, round (99.85%), textured, extra-high profile | Textured (POLYtxt) | 300–500 cc common; range: 250–835 | 250–835 |
| Góes et al. | 2003 | Retrospective Case series | 241 | Subfascial | Areolar or Inframammary | Silicone (McGhan 410 anatomical) | Textured | NS | NS |
| Graf et al. | 2000 | Retrospective Case series | 62 | Subfascial | Transaxillary (Endoscopic S-shape) | McGhan 410, anatomical, high-cohesivity silicone | Textured | NS | 155–235 g |
| Gryskiewicz and LeDuc | 2014 | Retrospective Case series | 2000 | Submuscular | Transaxillary | Saline (Phase 1), Gel or Saline (Phase 2) | Smooth | 354.4 ± 61.2 | 180–800 |
| Hammond et al. | 2012 | Prospective, Multicenter Clinical Trial | 572 | Submuscular | Inframammary (91.5%), Periareolar (5.9%), Other (2.6%) | Mentor Contour Profile Gel (MemoryShape, anatomical, cohesive silicone) | Textured | NS | NS |
| Han et al. | 2019 | Prospective Cohort Study | 52 | Dual plane | Transaxillary (74.8%), Inframammary (22.8%), Periareolar (2.4%) | BellaGel (smooth round) | Smooth (except one textured) | 232 | NS |
| Haws et al. | 2014 | Prospective, Multicenter Clinical Trial | 321 | Subglandular | Inframammary (66.9%), Periareolar (31.9%), Mastopexy (1.3%) | Sientra HSC+ Shaped Silicone Gel | Textured (TRUE Texture) | NS | NS |
| Haws et al. | 2015 | Prospective, Multicenter Clinical Trial Subanalysis | 935 | Submuscular (56%) vs Subglandular (44%) | Inframammary (64%), Periareolar (30%), Transaxillary (6%) | Sientra High-Strength Cohesive Silicone, Round and Shaped | Smooth (52%), Textured (48%) | 360 | 175–700 |
| Hetter et al. | 1991 | Retrospective Case series | 179 | Submuscular | Transaxillary | Silicone (McGhan double-lumen) | Not specified | 260 | 160–400 |
| Hidalgo et al. | 2000 | Retrospective Case series | 77 | Submuscular | Transaxillary (most common), Periareolar, Inframammary (selected cases) | Saline (majority), some Silicone | Smooth (most), Textured in select revisions | NS | NS |
| Huang et al. | 2011 | Retrospective Case series | 1682 | Submuscular | Transaxillary | Silicone (37.75%) and Saline (62.25%) | 99.8% Smooth, 0.2% Textured | 438.5 ± 51.5 | 270–630 |
| Huemer et al. | 2018 | Retrospective Case series | 100 | Dual plane | Inframammary | Silicone (Motiva Ergonomix) | SilkSurface (Nano-textured) | 370 | 150–700 |
| Hwang et al. | 2016 | Retrospective Case series | 30 | Dual plane | Inframammary | Cohesive gel (27 anatomical, 3 round) | Textured | Right: 288.9, Left: 281.6 | 255–360 |
| Jacobson et al. | 2012 | Retrospective Case series | 183 | Dual plane | Inframammary (115), Periareolar (44), Transaxillary (24) | Saline (70), Silicone (113) | Smooth (170), Textured (13) | NS | NS |
| Jewell et al. | 2010 | Prospective Cohort Study (Two Cohorts) | 236 | Allergan: 60% Subglandular vs 40% Dual plane vs Mentor: 40% Subglandular vs 60% Dual plane | Inframammary (100%) | Allergan Natrelle 410, Mentor CPG (form-stable cohesive gel) | Textured | NS | NS |
| Junior et al. | 2019 | Prospective Randomized Double-Blinded Study | 20 | Subfascial 50% vs Subglandular 50% | Inframammary | Round, textured silicone implants (Silimed) | Textured | NS | NS |
| Karabeg et al. | 2019 | Prospective Case Series | 27 | Dual plane | Inframammary (100%) | Mentor textured round silicone gel | Textured | 300 | 225–400 |
| Khan et al. | 2019 | Retrospective, Single-Surgeon 13-Year Series | 1511 | Dual plane (Type 2) | Inframammary (100%) | Round silicone gel (various manufacturers) | Textured (96.9%), Smooth (3.1%) | 340 | 170–700 |
| Khan et al. (2013) | 2013 | Retrospective, 12-Year Comparative Analysis | 2026 | Subglandular (39.1%) vs Submuscular (5.4%) vs Dual plane (Type 2) (55.4%) | Inframammary (majority) | Round cohesive silicone gel | Textured (predominantly) | SG: 317.5, SM: 300.6, BP: 338.9 | 170–655 |
| Khan et al. (2020) | 2020 | Retrospective Comparative Analysis | 2521 | Subglandular (32.7%), Submuscular (6.4%), Dual plane (60.8%) | Inframammary (100%) | Round silicone gel | Smooth (2.6%), Textured (44.8%), Microtextured (52.6%) | NS | 170–700 |
| Khoo et al. | 2016 | Retrospective Comparative Analysis | 416 | Subglandular | Periareolar (50.5%), Inframammary (49.5%) | Silimed cohesive polyurethane silicone gel | Polyurethane coated (textured equivalent) | 300.0 | NS |
| Kjøller et al. | 2001 | Retrospective Case series | 754 | Submuscular (90%) vs Subglandular (10%) | 70.2% Inframammary, 19.7% Periareolar | Silicone (single & double lumen), some saline and bio-gel | 52.3% Textured, 25.3% Smooth, remainder missing | 247 | 110–630 |
| Kolker et al. | 2010 | Retrospective Case series | 197 | Submuscular | Transaxillary | Saline (Smooth round) | Initially textured, then smooth exclusively | 298 | NS |
| Kooiman et al. | 2021 | prospective | 44 | Dual-plane (modified type 3) (50%) vs Subglandular (40%) | Inframammary | Eurosilicone Cristalline Paragel (round, micro-textured) | Micro-textured | 290.3 | Smooth: 180–605, Textured: 200–600 |
| Lee et al. | 2024 | Retrospective Case series | 70 | Dual plane | Transumbilical | Cohesive silicone gel (Mentor Smooth Round Moderate Plus) | Smooth | 272 | 225–300 |
| Lee et al. | 2012 | Retrospective Case series | 62 | Dual plane | Transareolar-perinipple (omega incision) | Smooth round cohesive silicone gel (Allergan) | Smooth | Right: 238.2, Left: 236.1 | 185–304 |
| Leite et al. | 2022 | Prospective Cohort Study | 20 | Submuscular | Inframammary | Round silicone-gel (Lifesil) | Microtextured | 270.0 | 175–325 |
| Lista et al. | 2013 | Retrospective Case Series | 440 | Subglandular | Inframammary | Allergan Style 410 (anatomic, highly cohesive silicone) | Textured | 385 | 215–775 |
| Lista et al. | 2019 | Retrospective Comparative Cohort Study | 248 | Subglandular | Inframammary or vertical (not explicitly separated for primary augmentation) | Smooth or Textured round silicone gel | Smooth (251), Textured (244) | Smooth: 373.6, Textured: 365.6 | Smooth: 180–605, Textured: 200–600 |
| Lonie et al. | 2023 | Retrospective Case Series (Single Surgeon) | 288 | Dual plane | Inframammary (100%) | Textured anatomic (98.6%), Round textured (1%), Round smooth (0.3%) | Textured | NS | NS |
| Luan et al. | 2009 | Prospective cohort | 49 | Dual plane | Transaxillary | Silicone (Allergan 410 anatomical) | Textured | NS | 185–315 |
| Marangi et al. | 2023 | Retrospective cohort | 71 | Dual plane | Inframammary or Inferior Hemi-Periareolar | Mentor silicone-filled (round and anatomical) | Microtextured | Median 375 | NS |
| Marchac et al. | 2021 | Prospective Multicenter Cohort | 653 | Submuscular | Inframammary (59.2%), Periareolar (32.3%) | SEBBIN Silicone Gel-Filled (Smooth, Semi-smooth, Microtextured, Macrotextured) | Smooth (0.6%), Semi-smooth (34.5%), Microtextured (26%), Macrotextured (39%) | 331.2 | NS |
| Maxwell et al. | 2021 | Retrospective Case Series (Single Site) | 777 | Submuscular | Inframammary or Lateral Areolar | Mentor MemoryGel Smooth Silicone | Smooth | 349.5 | 175–650 |
| Miller et al. | 2019 | Prospective Cohort | 34 | Submuscular | Inframammary | Silicone (Smooth round or Textured anatomic) | 24% Smooth, 76% Textured | 520 | 350–700 |
| Mirzabeigi et al. | 2012 | Retrospective Comparative Cohort | 257 | Dual plane | Inframammary and Periareolar | Mentor Smooth Silicone and Saline | Smooth | NS | NS |
| Montemurro et al. | 2023 | Retrospective Single-Surgeon Case Series | 1617 | Dual plane | Inframammary | Silicone | Textured (91.1%), Smooth (8.9%) | 305.37 | 140–615 |
| Montemurro et al. (2016) | 2016 | Retrospective Single-Surgeon Case Series | 310 | Dual plane | Inframammary | Allergan Style 410 Extra Full Projection Anatomical | Macrotextured | 338 | 195–615 |
| Montemurro et al. (2017) | 2017 | Retrospective Single-Surgeon Case Series | 648 | Dual plane | Inframammary | Silicone (Anatomical and Round) | Textured | 312 | 140–615 |
| Montemurro et al. (2024) | 2024 | Retrospective Single-Surgeon Case Series | 1212 | Dual plane | Inframammary | Silicone (Natrelle, POLYTECH, Motiva, Mentor) | Textured and Smooth | 316.09 | 145–615 |
| Munhoz et al. | 2024 | Retrospective cohort | 906 | Subfascial | Transaxillary | Silicone (various brands, round/shaped/ergonomic) | 68.9% Textured, 31.1% Smooth | 267.3 | 160–475 |
| Munhoz et al. | 2006 | Retrospective cohort | 42 | Subfascial | Transaxillary | Silicone gel (Silimed & McGhan) | Textured | NS | 210–305 |
| Namnoum et al. | 2013 | Prospective non-randomized clinical trial | 4412 | Submuscular vs Subglandular | Inframammary (87.1%), Periareolar (11.4%), Axillary (1.5%) | Silicone, Round or Anatomical | Smooth (6.1%), Textured (93.9%) | 325 | NS |
| Nguyen et al. | 2021 | Prospective Single-Surgeon Comparative Study | 275 | Dual plane | Endoscopic Transaxillary (205), Periareolar (70) | Round Smooth Silicone (Mentor, Style 4000) | Smooth | 275–300 most common | 200–350 |
| Nichter et al. | 2023 | Prospective Multicenter Clinical Trial – 10-Year Outcomes | 399 | Submuscular | Inframammary (70.8%), Periareolar (22.2%), Axillary (7.0%) | Ideal Implant (Double-lumen saline, structured) | Smooth | Varied – e.g., 335cc most commonly illustrated | 210–675 |
| Opitz et al. | 1998 | Retrospective Single-Surgeon Case Series | 86 | Subglandular | Periareolar or Axillary | Soybean Oil (Trilucent®) | Textured | 270 | 180–330 |
| Panettiere et al. | 2003 | Retrospective cohort | 469 | Submuscular | Inferior periareolar | 98.7% Silicone gel (round), 1.4% Anatomical | 63.4% Textured, 36.6% Smooth | 275 | 110–510 |
| Pelle-Ceravolo et al. | 2004 | Prospective cohort | 348 | Submuscular | Periareolar | Noncohesive gel-filled | 72.1% Polyurethane-covered, 27.9% Textured silicone | NS | NS |
| Pereira et al. | 2009 | Prospective cohort | 18 | Subfascial (33%) vs Subglandular (33%) vs Submuscular (33%) | Transaxillary | Silicone (textured) | Textured | NS | 190–300 |
| Puckett et al. | 1987 | Prospective cohort | 50 | Subglandular (50%) vs Submuscular (50%) | Inframammary | Silicone gel-filled (Dow Corning, low-profile round) | Not specified | 220 | NS |
| Rancati et al. | 2023 | Prospective Comparative Study | 50 | Subglandular | Inframammary (Central vs. Laterally displaced) | Silicone gel (Round) | Smooth | Cohort 1: 292.8 ± 46.2, Cohort 2: 283 ± 54.7 | 200–350 approx. |
| Randquist et al. | 2018 | Retrospective Chart Review | 22 | Dual plane | Inframammary | Form-stable anatomical silicone gel | Not stated | NS | NS |
| Riggio et al. | 2012 | Prospective Single-Surgeon Study | 50 | Dual plane | Inframammary (most), Areolar (few) | Silicone dual-gel (Natrelle 510) | Textured | 344.6 ± 57.9 | 130–495 |
| Seckel | 1993 | Retrospective cohort | 41 | Submuscular | inframammary | Silicone | smooth | NS | NS |
| Shi et al. | 2015 | Retrospective Comparative Study | 144 | Submuscular vs Subglandular | Axillary or Periareolar | Cohesive silicone gel | Textured | Subglandular: 201.9, Subpectoral: 199.6 | 175–335 |
| Short et al. | 2021 | Prospective Observational Study (Post-Approval) | 6743 | Submuscular | Not specified | Mentor MemoryGel | Smooth (91.2%), Textured (8%) | NS | NS |
| Siclovan et al. | 2008 | Retrospective cohort | 45 | Subfascial | Inframammary | Silicone gel (Mentor CPG 300 anatomical) | Textured | NS | 225–300 (based on case examples) |
| Sim et al. | 2018 | Prospective Case Series | 76 | Subfascial | Transaxillary | Silicone (Motiva Ergonomix) | Microtextured (SilkSurface) | 278.1 | 185–360 |
| Sohn et al. | 2000 | retrospective cohort | 19 | Submuscular | Periareolar (4–8 o’clock) | Hydrogel | Not specified | NS | 140–220 |
| Stan et al. | 2017 | Retrospective Single-Surgeon Series | 763 | Dual plane | Inframammary only | Silicone (Diagon/Gel) | Micropolyurethane foam (Microthane) | 270 | NS |
| Swanson et al. | 2020 | Retrospective Cohort Study | 145 | Submuscular | Mostly supra-inframammary | Mentor smooth, round, Moderate Plus Profile saline | Smooth | 424 | 270–800 |
| Tijerina et al. | 2009 | Retrospective | 1000 | Subfascial | 95% inframammary , 5% Periareolar | Cohesive soft gel silicone | Textured | 310 | 180-600 |
| Tanner et al. | 2017 | Retrospective Single-Surgeon Series | 126 | Subfascial | Inframammary | Silicone (Groupe Sebbin LS 90/LSC 92/LSC 93) | Microtextured | 337.8 | NS |
| Vazquez et al. | 1987 | Retrospective Cohort | 98 | Submuscular vs Subglandular | Inferior circumareolar | Silicone gel (various manufacturers) | Smooth (mostly) | NS | NS |
| Venkataram et al. | 2023 | Prospective | 2088 | Dual plane | Inframammary | Silicone | Smooth | NS | NS |
| Wieslander et al. | 2020 | Retrospective | 1310 | Submuscular | Axillary (2.5–3.5 cm) | Perthese textured round cohesive silicone gel | Textured | 300–400 (most common range) | NS |
| Ya et al. | 2024 | Retrospective | 82 | Dual plane | Transaxillary | Mentor microtextured round or anatomical | Microtextured | 272.5 | NS |
| Zaussinger et al. | 2023 | Retrospective | 340 | Dual plane | Inframammary | Nagor Impleo round silicone gel | Textured | 390 | 240–560 |

Table , SDC 1
