# Supplementary material for: Implant Pocket Plane Selection in Primary Breast Augmentation: A Meta-Analysis and Systematic Review of Complication Profiles
Source: Aesthetic Plast Surg. 2026 Feb 26;50(11):3979–87. doi: 10.1007/s00266-026-05706-5 (PMC13315495; doi:10.1007/s00266-026-05706-5)
Supplement: Supplementary file 2 — Supplementary file2 (DOCX 25 kb) [file 266_2026_5706_MOESM2_ESM.docx]

**Plane Selection in Primary Breast Augmentation – Referenced Papers:**

1. Aboelatta YA, Aboelatta H, Elgazzar K. A simple method for proper placement of the inframammary fold incision in primary breast augmentation. *Ann Plast Surg*. 2015;75(5):497-502. doi:10.1097/SAP.0000000000000158
2. Adams WP Jr. The process of breast augmentation: four sequential steps for optimizing outcomes for patients. *Plast Reconstr Surg*. 2008;122(6):1892-1900. doi:10.1097/PRS.0b013e31818d20ec
3. Adams WP Jr, Rios JL, Smith SJ. Enhancing patient outcomes in aesthetic and reconstructive breast surgery using triple antibiotic breast irrigation: six-year prospective clinical study. *Plast Reconstr Surg*. 2006;117(1):30-36. doi:10.1097/01.prs.0000185671.51993.7e
4. Alderman A, Caplin D, Hammond DC, Keane A, Turetzky J, Kane WJ. Clinical results of Mentor MemoryGel Xtra breast implants from the GLOW clinical trial. *Aesthet Surg J*. 2023;43(12):NP1021-NP1032. doi:10.1093/asj/sjad272
5. Araco A, Gravante G, Araco F, et al. A retrospective analysis of 3,000 primary aesthetic breast augmentations: postoperative complications and associated factors. *Aesthetic Plast Surg*. 2007;31(5):532-539. doi:10.1007/s00266-007-0162-8
6. Asplund O, Gylbert L, Jurell G, Ward C. Textured or smooth implants for submuscular breast augmentation: a controlled study. *Plast Reconstr Surg*. 1996;97(6):1200-1206.
7. Aygit AC, Basaran K, Mercan ES. Transaxillary totally subfascial breast augmentation with anatomical breast implants: review of 27 cases. *Plast Reconstr Surg*. 2013;131(5):1149-1156. doi:10.1097/PRS.0b013e3182865d68
8. Barbato C, Pena M, Triana C, Zambrano MA. Augmentation mammoplasty using the retrofascia approach. *Aesthetic Plast Surg*. 2004;28(3):148-152. doi:10.1007/s00266-004-4014-5
9. Batiukov D, Podgaiski V. Nonadherence of polyurethane implants: a retrospective cohort study. *Indian J Plast Surg*. 2024;57(1):24-30. doi:10.1055/s-0043-1778644
10. Bengtson BP, Van Natta BW, Murphy DK, Slicton A, Maxwell GP; Style 410 U.S. Core Clinical Study Group. Style 410 highly cohesive silicone breast implant core study results at 3 years. *Plast Reconstr Surg*. 2007;120(7 Suppl 1):40S-48S. doi:10.1097/01.prs.0000286666.29101.11
11. Bletsis PP, van der Lei B. Key insights from a decade of breast augmentation: our 5 critical decisions in breast implant selection. *Plast Reconstr Surg Glob Open*. 2025;13(4):e6695. doi:10.1097/GOX.0000000000006695
12. Blount AL, Martin MD, Lineberry KD, Kettaneh N, Alfonso DR. Capsular contracture rate in a low-risk population after primary augmentation mammaplasty. *Aesthet Surg J*. 2013;33(4):516-521. doi:10.1177/1090820X13484465
13. Bolletta A, Dessy LA, Fiorot L, et al. Sub-muscular breast augmentation using tumescent local anesthesia. *Aesthetic Plast Surg*. 2019;43(1):7-13. doi:10.1007/s00266-018-1181-3
14. Bosch G, Jacobo O. The double pocket technique: aesthetic breast augmentation. *Aesthetic Plast Surg*. 2002;26(6):461-464. doi:10.1007/s00266-002-1485-0
15. Brown MH, Shenker R, Silver SA. Cohesive silicone gel breast implants in aesthetic and reconstructive breast surgery. *Plast Reconstr Surg*. 2005;116(3):768-779. doi:10.1097/01.prs.0000176259.66948.e7
16. Brown T. Subfascial breast augmentation: is there any advantage over the submammary plane? *Aesthetic Plast Surg*. 2012;36(3):566-569. doi:10.1007/s00266-011-9840-7
17. Bruck HG. Long-term results of polyurethane-covered prostheses. *Aesthetic Plast Surg*. 1990;14(1):85-86. doi:10.1007/BF01578331
18. Chatterjee SS, Khanna M. Hundred and eleven cases of subfascial breast augmentation in trans women—a single-center experience. *Indian J Plast Surg*. 2020;53(1):28-35. doi:10.1055/s-0040-1708227
19. Coleman DJ, Foo IT, Sharpe DT. Textured or smooth implants for breast augmentation? A prospective controlled trial. *Br J Plast Surg*. 1991;44(6):444-448.
20. Dancey A, Nassimizadeh A, Levick P. Capsular contracture—what are the risk factors? A 14 year series of 1400 consecutive augmentations. *J Plast Reconstr Aesthet Surg*. 2012;65(2):213-218. doi:10.1016/j.bjps.2011.09.011
21. Diaz JF. Review of 494 consecutive breast augmentation patients: system to improve patient outcomes and satisfaction. *Plast Reconstr Surg Glob Open*. 2017;5(10):e1526. doi:10.1097/GOX.0000000000001526
22. Fanous N, Salem I, Tawilé C, Bassas AE. Absence of capsular contracture in 319 consecutive augmentation mammaplasties: dependent drains as a possible factor. *Can J Plast Surg*. 2004;12(4):193-197.
23. Góes JC, Landecker A. Optimizing outcomes in breast augmentation: seven years of experience with the subfascial plane. *Aesthetic Plast Surg*. 2003;27(3):178-184. doi:10.1007/s00266-003-0004-2
24. Chiemi JA, Kelishadi SS. A Rationale for Micro-textured Breast Implant Augmentation. Aesthetic Surgery Journal Open Forum. Published online March 30, 2022. doi:https://doi.org/10.1093/asjof/ojac020
25. Govrin-Yehudain O, Calderon N, Govrin-Yehudain J. Five-year safety and satisfaction with the lightweight breast implant. *Aesthet Surg J*. 2021;41(10):NP1362-NP1373. doi:10.1093/asj/sjab054
26. Graf RM, Bernardes A, Auersvald A, Damasio RC. Subfascial endoscopic transaxillary augmentation mammaplasty. *Aesthet Plast Surg*. 2000;24(3):216-220. doi:10.1007/s002660010036
27. Gryskiewicz J, LeDuc R. Transaxillary nonendoscopic subpectoral augmentation mammaplasty: a 10-year experience with gel vs saline in 2000 patients—with long-term patient satisfaction measured by the BREAST-Q. *Aesthet Surg J*. 2014;34(5):696-713. doi:10.1177/1090820X14530552
28. Hammond DC, Migliori MM, Caplin DA, Garcia ME, Phillips CA. Mentor Contour Profile Gel implants: clinical outcomes at 6 years. *Plast Reconstr Surg*. 2012;129(6):1381-1391. doi:10.1097/PRS.0b013e31824ecbf0
29. Han J, Jeong JH, Bang SI, Heo CY. BellaGel breast implant: 4-year results of a prospective cohort study. *J Plast Surg Hand Surg*. 2019;53(5):297-305. doi:10.1080/2000656X.2019.1583572
30. Haws MJ, Alizadeh K, Kaufman DL. Sientra primary and revision augmentation rupture trending and analysis with magnetic resonance imaging. *Aesthet Surg J*. 2015;35(Suppl 1):S33-S42. doi:10.1093/asj/sjv021
31. Haws MJ, Schwartz MR, Berger LH, Daulton KL. Sientra portfolio of Silimed brand shaped implants with high-strength silicone gel: a 5-year primary augmentation clinical study experience and a postapproval experience—results from a single-surgeon 108-patient series. *Plast Reconstr Surg*. 2014;134(3 Suppl):38S-46S. doi:10.1097/PRS.0000000000000346
32. Hetter GP. Improved patient satisfaction with augmentation mammoplasty: the transaxillary subpectoral approach. *Aesthet Plast Surg*. 1991;15(2):123-127. doi:10.1007/BF02273846
33. Hidalgo DA. Breast augmentation: choosing the optimal incision, implant, and pocket plane. *Plast Reconstr Surg*. 2000;105(6):2202-2216; discussion 2217-2218. doi:10.1097/00006534-200005000-00047
34. Huang GJ, Wichmann JL, Mills DC. Transaxillary subpectoral augmentation mammaplasty: a single surgeon's 20-year experience. *Aesthet Surg J*. 2011;31(7):781-801. doi:10.1177/1090820X11416936
35. Huemer GM, Wenny R, Aitzetmüller MM, Duscher D. Motiva Ergonomix round SilkSurface silicone breast implants: outcome analysis of 100 primary breast augmentations over 3 years and technical considerations. *Plast Reconstr Surg*. 2018;141(6):831e-842e. doi:10.1097/PRS.0000000000004367
36. Hwang DY, Park SH, Kim SW. A modified dual-plane technique using the serratus anterior fascia in primary breast augmentation. *Plast Reconstr Surg Glob Open*. 2017;5(2):e1213. doi:10.1097/GOX.0000000000001213
37. Jacobson JM, Gatti ME, Schaffner AD, Hill LM, Spear SL. Effect of incision choice on outcomes in primary breast augmentation. *Aesthet Surg J*. 2012;32(4):456-462. doi:10.1177/1090820X12444267
38. Jewell ML, Jewell JL. A comparison of outcomes involving highly cohesive, form-stable breast implants from two manufacturers in patients undergoing primary breast augmentation. *Aesthet Surg J*. 2010;30(1):51-65. doi:10.1177/1090820X09360700
39. Karabeg R, Jakirlic M, Karabeg A, Crnogorac D, Aslani I. The new method of pocket forming for breast implant placement in augmentation mammaplasty: dual plane subfascial. *Med Arch*. 2019;73(3):178-182. doi:10.5455/medarh.2019.73.178-182
40. Maluf Junior I, Graf RM, Ascenço ASK, et al. Is there a breast augmentation outcome difference between subfascial and subglandular implant placement? A prospective randomized double-blinded study. *Aesthet Plast Surg*. 2019;43(6):1429-1436. doi:10.1007/s00266-019-01465-8
41. Khan UD. Muscle-splitting, subglandular, and partial submuscular augmentation mammoplasties: a 12-year retrospective analysis of 2026 primary cases. *Aesthet Plast Surg*. 2013;37(2):290-302. doi:10.1007/s00266-012-0026-8
42. Khan UD. Muscle splitting augmentation mammoplasty: a 13-year outcome analysis of 1511 primary augmentation mammoplasties. *Aesthet Plast Surg*. 2019;43(6):1499-1506. doi:10.1007/s00266-019-01468-5
43. Khan UD. Periprosthetic infection in primary and secondary augmentation mammoplasty using round silicone gel breast implants: comparative analysis of 2521 primary and 386 secondary mammoplasties in a single surgeon practice. *Aesthet Plast Surg*. 2021;45(1):1-10. doi:10.1007/s00266-020-01965-y
44. Khoo LS, Radwanski HN, Senna-Fernandes V, Antônio NN, Fellet LLF, Pitanguy I. Does the use of intraoperative breast sizers increase complication rates in primary breast augmentation? A retrospective analysis of 416 consecutive cases in a single institution. *Plast Surg Int*. 2016;2016:6584810. doi:10.1155/2016/6584810
45. Kjøller K, Hölmich LR, Jacobsen PH, et al. Capsular contracture after cosmetic breast implant surgery in Denmark. *Ann Plast Surg*. 2001;47(4):359-366.
46. Kolker AR, Austen WG Jr, Slavin SA. Endoscopic-assisted transaxillary breast augmentation: minimizing complications and maximizing results with improvements in patient selection and technique. *Ann Plast Surg*. 2010;64(5):667-673. doi:10.1097/SAP.0b013e3181d9aa3d
47. Kooiman LBR, Torensma B, Stevens HPJD, van der Lei B. Single center and surgeon's long-term (15-19 years) patient satisfaction and revision rate of round textured Eurosilicone breast implants. *Aesthet Surg J*. 2022;42(5):NP282-NP292. doi:10.1093/asj/sjab373
48. Lee JH, Lee PK, Oh DY, Rhie JW, Ahn ST. Subpectoral-subfascial breast augmentation for thin-skinned patients. *Aesthet Plast Surg*. 2012;36(1):115-121. doi:10.1007/s00266-011-9761-5
49. Lee W, Kim SH. Transumbilical silicone breast augmentation: a 13-year experience and 5-year analysis. *Aesthet Plast Surg*. 2024;48(18):3627-3636. doi:10.1007/s00266-024-03850-4
50. Leite AT, Sabino-Neto M, Lopes Resende VC, Veiga DF, Ferreira LM. Patient-reported outcomes after subpectoral breast augmentation with microtextured or macrotextured implants using the BREAST-Q. *Arch Plast Surg*. 2022;49(3):352-359. doi:10.1055/s-0042-1748649
51. Lista F, Austin RE, Saheb-Al-Zamani M, Ahmad J. Does implant surface texture affect the risk of capsular contracture in subglandular breast augmentation and breast augmentation-mastopexy? *Aesthet Surg J*. 2020;40(5):499-506. doi:10.1093/asj/sjz241
52. Lista F, Tutino R, Khan A, Ahmad J. Subglandular breast augmentation with textured, anatomic, cohesive silicone implants: a review of 440 consecutive patients. *Plast Reconstr Surg*. 2013;132(2):295-303. doi:10.1097/PRS.0b013e3182958a6d
53. Lonie S, Abesamis GM, Law J, et al. Topical tranexamic acid in primary breast augmentation surgery: short- and long-term outcomes. *Aesthet Surg J*. 2024;44(1):NP23-NP27. doi:10.1093/asj/sjad219
54. Luan J, Mu D, Mu L. Transaxillary dual-plane augmentation mammaplasty: experience with 98 breasts. *J Plast Reconstr Aesthet Surg*. 2009;62(11):1459-1463. doi:10.1016/j.bjps.2008.05.044
55. Marangi GF, Gratteri M, Mirra C, et al. The correlation between the improvement in patient's quality of life and surgeon's satisfaction following primary breast augmentation: a prospective study. *Aesthet Surg J*. 2023;43(7):741-747. doi:10.1093/asj/sjad052
56. Marchac A, El Haddad R, Boedec C, et al. Three-year intermediate results of a prospective multicenter study investigating the use of smooth, semi-smooth, microtextured and macrotextured implants from a single manufacturer in breast augmentation and reconstruction procedures. *J Plast Reconstr Aesthet Surg*. 2021;74(12):3325-3332. doi:10.1016/j.bjps.2021.01.020
57. Maxwell D, Estes M, McMillen Walcott J, et al. Safety and effectiveness of silicone gel-filled breast implants in primary augmentation patients. *Aesthet Surg J*. 2021;41(6):NP329-NP335. doi:10.1093/asj/sjaa388
58. Miller TJ, Wilson SC, Massie JP, Morrison SD, Satterwhite T. Breast augmentation in male-to-female transgender patients: technical considerations and outcomes. *JPRAS Open*. 2019;21:63-74. doi:10.1016/j.jpra.2019.03.003
59. Mirzabeigi MN, Mericli AF, Ortlip T, et al. Evaluating the role of postoperative prophylactic antibiotics in primary and secondary breast augmentation: a retrospective review. *Aesthet Surg J*. 2012;32(1):61-68. doi:10.1177/1090820X11430830
60. Montemurro P, Cheema M, Hedén P, Agko M, Quattrini Li A, Avvedimento S. Do not fear an implant's shape: a single surgeon's experience of over 1200 round and shaped textured implants in primary breast augmentation. *Aesthet Surg J*. 2018;38(3):254-261. doi:10.1093/asj/sjx145
61. Montemurro P, Cheema M, Hedén P, Ferri M, Quattrini Li A, Avvedimento S. Role of macrotextured shaped extra full projection cohesive gel implants in primary aesthetic breast augmentation. *Aesthet Surg J*. 2017;37(4):408-418. doi:10.1093/asj/sjw199
62. Montemurro P, Gupta T. It is time to resolve the dilemma and move away from using drains in primary breast augmentation. *Aesthet Surg J Open Forum*. 2023;5:ojad048. doi:10.1093/asjof/ojad048
63. Montemurro P, Pietruski P. Twelve years and over 2400 implants later: augmentation mammoplasty risk factors based on a single plastic surgeon's experience. *Plast Reconstr Surg Glob Open*. 2024;12(4):e5720. doi:10.1097/GOX.0000000000005720
64. Munhoz AM, Fells K, Arruda E, et al. Subfascial transaxillary breast augmentation without endoscopic assistance: technical aspects and outcome. *Aesthetic Plast Surg*. 2006;30(5):503-512. doi:10.1007/s00266-006-0017-8
65. Munhoz AM, Marques Neto AA. Subfascial transaxillary breast augmentation: critical evaluation of a 25-year review of 1015 consecutive cases. *Plast Reconstr Surg*. 2025;155(2):462-476. doi:10.1097/PRS.0000000000011612
66. Namnoum JD, Largent J, Kaplan HM, Oefelein MG, Brown MH. Primary breast augmentation clinical trial outcomes stratified by surgical incision, anatomical placement and implant device type. *J Plast Reconstr Aesthet Surg*. 2013;66(9):1165-1172. doi:10.1016/j.bjps.2013.04.046
67. Nguyen HH, To LT. Comparison of endoscopic transaxillary and peri-areolar approaches in breast augmentation with smooth implants. *Aesthet Plast Surg*. 2021;45(5):2101-2110. doi:10.1007/s00266-021-02448-4
68. Nichter LS, Hardesty RA, Zimmerman TJ. Ideal Implant structured breast implants: core study results through 10 years. *Plast Reconstr Surg*. 2023;152(3):424e-432e. doi:10.1097/PRS.0000000000010312
69. Opitz PG, Young VL. Experience with soybean oil-filled breast implants in a Swedish surgical practice. *Aesthet Surg J*. 1998;18(3):183-188.
70. Panettiere P, Marchetti L, Accorsi D, Del Gaudio GA. Augmentation mammaplasty of teardrop-shaped breasts using round prostheses. *Aesthet Plast Surg*. 2003;27(1):20-26. doi:10.1007/s00266-002-0061-y
71. Pelle-Ceravolo M, Del Vescovo A, Bertozzi E, Molinari P. A technique to decrease breast shape deformity during muscle contraction in submuscular augmentation mammaplasty. *Aesthet Plast Surg*. 2004;28(5):288-294. doi:10.1007/s00266-003-3023-0
72. Pereira LH, Sterodimas A. Transaxillary breast augmentation: a prospective comparison of subglandular, subfascial, and submuscular implant insertion. *Aesthet Plast Surg*. 2009;33(5):752-759. doi:10.1007/s00266-009-9389-x
73. Puckett CL, Croll GH, Reichel CA, Concannon MJ. A critical look at capsule contracture in subglandular versus subpectoral mammary augmentation. *Aesthetic Plast Surg*. 1987;11(1):23-28.
74. Randquist C, Por YC, Yeow V, Maglambayan J, Simonyi S. Breast augmentation surgery using an inframammary fold incision in Southeast Asian women: Patient-reported outcomes. *Arch Plast Surg*. 2018;45(4):367-374. doi:10.5999/aps.2018.00045
75. Rancati AO, Nahabedian MY, Angrigiani C, Dip F, Dorr J, Rancati A. Sensory evaluation of the nipple-areolar complex following primary breast augmentation: a comparison of incision approaches. *Aesthet Surg J*. 2023;43(12):NP1013-NP1020. doi:10.1093/asj/sjad276
76. Riggio E. Breast augmentation with extra-projected and high-cohesive dual-gel Prosthesis 510: a prospective study of 75 consecutive cases for a new method (the Zenith System). *Aesthet Plast Surg*. 2012;36(4):866-878. doi:10.1007/s00266-012-9889-y
77. Seckel B, Costas PD. Total versus partial musculofascial coverage for steroid-containing double-lumen breast implants in augmentation mammaplasty. *Ann Plast Surg*. 1993;30(4):296-303.
78. Shi H, Cao C, Li X, Chen L, Li S. A retrospective study of primary breast augmentation: recovery period, complications and patient satisfaction. *Int J Clin Exp Med*. 2015;8(10):18737-18743.
79. Short KK, Wixtrom RN, Estes MM, Leopold J, Canady JW. Results from the MemoryGel Post-approval Study. *Plast Reconstr Surg Glob Open*. 2021;9(3):e3402. doi:10.1097/GOX.0000000000003402
80. Sim HB. Revisiting prepectoral breast augmentation: indications and refinements. *Aesthet Surg J*. 2019;39(5):NP113-NP122. doi:10.1093/asj/sjy294
81. Siclovan HR, Jomah JA. Advantages and outcomes in subfascial breast augmentation: a two-year review of experience. *Aesthet Plast Surg*. 2008;32(3):426-431. doi:10.1007/s00266-008-9141-y
82. Sohn BK, Chung YJ, Kim G, Yoon WJ. Submuscular periareolar approach to augmentation mammoplasty in Korean women. *Aesthetic Plast Surg*. 2000;24(6):455-460. doi:10.1007/s002660010077
83. Stan C, Biggs T. The Diagon/Gel implant: a preliminary report of 894 cases. *Plast Reconstr Surg Glob Open*. 2017;5(7):e1393. doi:10.1097/GOX.0000000000001393
84. Swanson E. Prospective study of saline versus silicone gel implants for subpectoral breast augmentation. *Plast Reconstr Surg Glob Open*. 2020;8(6):e2882. doi:10.1097/GOX.0000000000002882
85. Tanner NSB. Low rate of capsular contracture in a series of 214 consecutive primary and revision breast augmentations using microtextured implants. *JPRAS Open*. 2017;14:66-73. doi:10.1016/j.jpra.2017.10.007
86. Tijerina VNE, Saenz RAE, Garcia-Guerrero J. Experience of 1000 cases on subfascial breast augmentation. *Aesthet Plast Surg*. 2010;34(1):16-22. doi:10.1007/s00266-009-9402-4
87. Vazquez B, Given KS, Houston GC. Breast augmentation: a review of subglandular and submuscular implantation. *Aesthetic Plast Surg*. 1987;11(2):101-105.
88. Venkataram A, Lahar N, Adams WP Jr. Enhancing patient outcomes in aesthetic breast implant procedures using proven antimicrobial breast pocket irrigations: a 20-year follow-up. *Aesthet Surg J*. 2023;43(1):66-73. doi:10.1093/asj/sjac238
89. Wieslander JB. Primary breast augmentation using axillary skin incision, submuscular implants, and intraoperative tissue expansion. *Plast Reconstr Surg Glob Open*. 2020;8(5):e2825. doi:10.1097/GOX.0000000000002825
90. Ya Z, Xiao L, Zhou L. The reverse dual plane: a novel technique for endoscopic transaxillary breast augmentation. *Aesthet Surg J Open Forum*. 2024;6:ojae020. doi:10.1093/asjof/ojae020
91. Zaussinger M, Duscher D, Huemer GM. Nagor Impleo round silicone gel breast implants: early outcome analysis after 340 primary breast augmentations. *J Clin Med*. 2023;12(11):3708. doi:10.3390/jcm12113708
